# Implementation Science Workshop: a Novel Multidisciplinary Primary Care Program to Improve Care and Outcomes for Super-Utilizers

**DOI:** 10.1007/s11606-016-3598-1

**Published:** 2016-03-28

**Authors:** Colleen S. Lynch, Ania Wajnberg, Ramiro Jervis, Maria Basso-Lipani, Susan Bernstein, Claudia Colgan, Theresa Soriano, Alex D. Federman, Sunil Kripalani

**Affiliations:** San Francisco Department of Public Health, San Francisco, CA USA; Division of General Internal Medicine, Icahn School of Medicine at Mount Sinai, New York, NY USA; Department of Social Work Services, Mount Sinai Medical Center, New York, NY USA; Section of Hospital Medicine, Division of General Internal Medicine and Public Health, Department of Medicine, Vanderbilt University Medical Center, Nashville, TN USA; Center for Clinical Quality and Implementation Research, Vanderbilt University Medical Center, Nashville, TN USA

**Keywords:** utilization, primary care redesign, vulnerable populations

## CASE

### Introduction

Patients with very high levels of hospitalizations and emergency room visits, sometimes identified as “super-utilizers,”^1^ often represent a population with unmet healthcare needs. Though patients in this group are heterogeneous and difficult to define across settings,^2^ they frequently have poor access to care, complex medical and social problems, and high healthcare costs. Programmatic interventions that target this group share the goal of improving care quality, and often employ care management to address the myriad psychosocial factors that complicate care, such as poor housing conditions, poverty, substance abuse, and mental illness.^3^–^7^ However, variations in design, focus, and setting among these longitudinal programs make comparisons challenging. As a result, the literature has yet to identify specific best practices for broad application.^8^

In 2010, the Mount Sinai Medical Center created the Preventable Admissions Care Team (PACT) Clinic to improve care for super-utilizers. By taking a team-based, high continuity and high intensity approach to primary care, we have created a promising care model for this high-risk group.

### Setting and Participants

The PACT Clinic is part of the Mount Sinai Hospital (MSH), a large tertiary teaching hospital situated in East Harlem, New York City. The clinic was formed in 2010 in order to receive referrals from an inpatient transitional care program built to reduce 30-day hospital readmission rates. Patients are identified for the transitional program if they score highly on an internally generated readmission risk score on inpatient admission; the score is based on the Hierarchical Condition Category score from the Center for Medicare and Medicaid Services^9^ and previous hospital utilization.

The PACT Clinic is located in the same space as Mount Sinai’s academic primary care practice, the Internal Medicine Associates (IMA); this co-location helps to improve care of complex patients and support resident medical education. The IMA clinic serves over 15,000 patients annually, is the teaching site for approximately 120 residents in internal medicine, and is the practice site of 30 attending-level clinicians. Patients seen at IMA often have a high burden of medical disease, many psychosocial stressors, and low health literacy, which complicates care delivery in a setting with finite resources for non-clinical needs. The complex scheduling of clinic sessions for medical residents in IMA has also contributed to the challenge of delivering care to this population by limiting provider continuity, a driver of satisfaction among both patients and primary care residents.^10^,^11^ Program leaders reasoned that removing the highest utilizers from the resident panels would improve access and quality of care for this vulnerable group, while allowing residents to have greater continuity with their remaining panel of patients.

### Program Description

#### Referral

**Figure 1:**
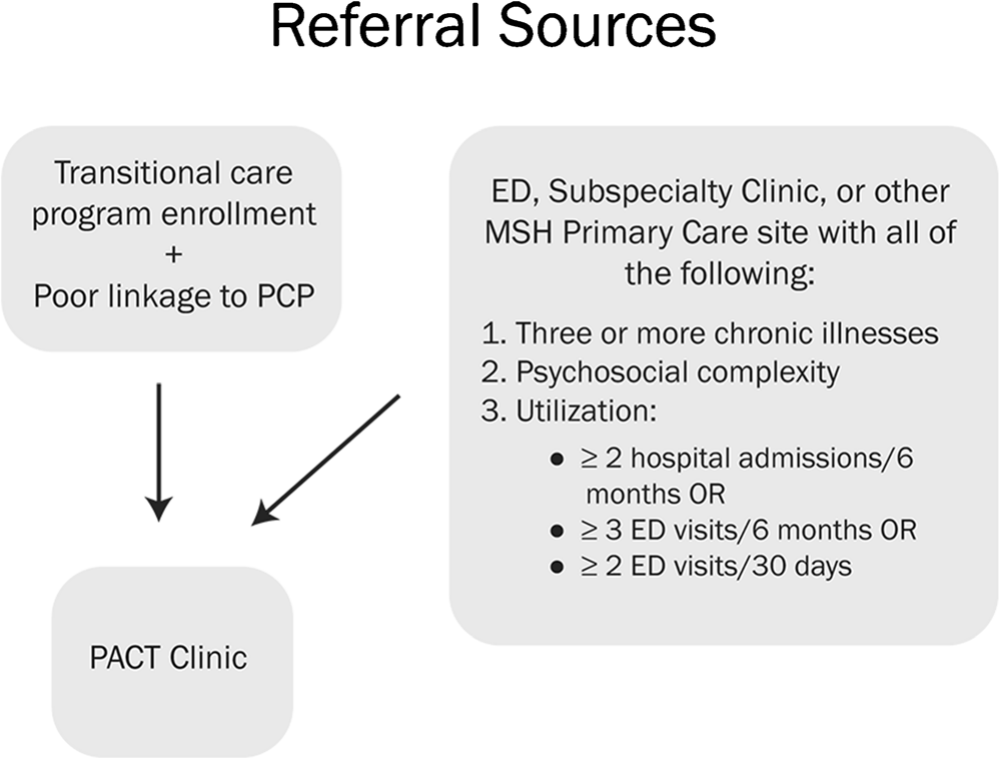
Referral sources for PACT clinic.

The PACT Clinic accepts patients from several sites in the MSH system, and referral criteria differ somewhat depending on the referral source. Referrals from subspecialty practices, primary care sites, or the Emergency Department (ED) should have three or more chronic illnesses, psychosocial complexity (e.g., low income, low health literacy, housing instability, substance abuse or psychiatric comorbidities), as well as one of the following utilization criteria: two or more hospitalizations in 6 months, three or more ED visits in 6 months, or two or more ED visits in 30 days. Patients in the post discharge transitional care program are all high risk, and so are referred to PACT Clinic directly if they lack a primary care provider or if they wish to transition to a new provider. See Fig. 1.

#### Clinical Teams

The PACT Clinic model pairs either a physician or a nurse practitioner with a social worker to follow patients together; currently six teams are active. The clinician member of the team provides primary medical services for a panel of up to 100 patients, enabling high provider continuity. These low ratios also allow for close collaboration between social workers and clinicians, as well as between the physicians and nurse practitioners, leveraging strengths of all members of the team. As the program has grown, it has expanded to include a front desk registrar, a medical assistant, and a dedicated administrative assistant to allow for focused recruitment, scheduling, tracking, and direction of patient phone calls to staff members.

Intensive social work support is another critical aspect of the PACT Clinic. The team social worker completes the care unit with the clinician, and the patients are seen by their social worker at every scheduled clinician visit. This focus on social work allows for rapid identification and intervention of psychosocial barriers to optimal health, including counseling for mental health problems, chronic disease self-management support, behavioral activation, insurance navigation, and linkage to support services. This team structure supports a high degree of care coordination and individualization; during weekly meetings, team members strategize to address patient challenges and goal setting.

#### Patient Scheduling

Open access is another distinctive feature of the PACT Clinic. The goal is to have up to one-third of the Doctor of Medicine (MD) and nurse practitioner (NP) daily visits reserved for walk-in or urgent appointments. All new patients are scheduled for a 1-h visit with a primary care provider and an additional hour with social work; follow-up appointments are 30 min for both MD/NP and social work. Each clinician reserves one half-session per week for home visits in order to enhance access for patients who are temporarily unable to come to clinic or when a home visit may provide information about care supports, medication adherence, or home environment. The smaller panel load also allows clinicians to meet patients in other locations, such as the ED, when necessary to assist in care planning. Most patients in PACT Clinic see a provider at least eight times in the first year of enrollment.

### Program Evaluation

#### Analysis

We reviewed the charts of patients enrolled in the PACT Clinic between November 2012 and October 2013. MSH electronic medical records were used for data on patient demographics, insurance, comorbidities, and service utilization. We compared acute care utilization at MSH before and after program enrollment. Using the date of PACT Clinic enrollment as the transition point, mean admission rates were calculated in the 6- or 12-month period before enrollment and compared to post-enrollment rates. Pre- and post-enrollment differences were tested for significance with the paired Wilcoxon rank sum test.

#### Results

From November 2012 to October 2013, 171 patients were enrolled in the PACT Clinic. Baseline clinical characteristics are shown in Table 1. In general, PACT Clinic patients were under-represented minorities and socioeconomically disadvantaged. For example, nearly all patients had Medicare, Medicaid, or were dually enrolled, and the median household income in the most common zip codes of residence was $31,254 and $26,214, respectively.^12^ Patients had a high burden of medical comorbidities with a mean number of seven (range 2–18). The group also had a significant burden of psychiatric illness, with depression, chronic anxiety, and schizophrenia being the most common. Substance abuse affected 14 %, and 134 (79 %) had received the PACT transitions intervention.

**Table 1.** Characteristics of IMA-PACT Patients at Enrollment From September 2012 to October 2013

	*N* = 171
Age (mean, SD)	63 (14)
Female	107 (63 %)
Ethnicity/race	
Black	84 (49 %)
Hispanic	79 (46 %)
Other	8 (5 %)
Insurance	
Medicare	43 (25 %)
Medicaid	51 (30 %)
Dual	77 (45 %)
Zip Code of Residence	
10029	53 (31 %)
10035	24 (14 %)
Comorbidities (mean, SD)	7 (4)
Hypertension	127 (74 %)
Diabetes Mellitus	93 (54 %)
COPD or Asthma	56 (33 %)
Chronic Kidney Disease	54 (32 %)
Heart Failure	52 (30 %)
Coronary Artery Disease	49 (29 %)
Chronic Pain	42 (25 %)
Depression or Bipolar	25 (15 %)
Substance Abuse ^*^	24 (14 %)
Unstable Housing^†^	27 (16 %)
Low Health Literacy^†^	51 (30 %)
Unpartnered^†^^,‡^	136 (80 %)

^*^Active; includes alcohol, cocaine, or heroin

^†^Obtained from social work evaluation

^‡^Single, widowed, divorced or separated

Mean hospital admission and ED visit rates generally fell after enrollment. For the 94 patients that met utilization enrollment criteria (Table 2), at 6 months post-PACT Clinic enrollment, mean hospitalization fell from 2.4 to 1.1 (*p* < 0.01) and ED visit rates fell from 1.6 to 1.2 (*p* = 0.03). Reductions in hospital utilization were persistent at 12 months, where the mean number of hospital admissions was reduced from 3.5 to 1.9 (*p* < 0.01); reductions seen in ED visits were not statistically significant (*p* = 0.14) .

**Table 2.** Mean Utilization Rates of Those Meeting Utilization Enrollment Criteria^*^ From Enrollment Date (September 2012 to October 2013) *N* = 94

Time	Location	Pre- Enrollment Mean (SD)	Post-Enrollment Mean (SD)	Percent Reduction	*p* value^†^
6 months	Hospital Admissions	2.4 (1.7)	1.1 (1.4)	54 %	< **0.01**
	Emergency Visits	1.6 (2.5)	1.2 (2.2)	25 %	**0.03**
12 months	Hospital Admissions	3.5 (2.9)	1.9 (2.3)	46 %	< **0.01**
	Emergency Visits	2.6 (4.1)	2.2 (3.6)	15 %	0.14

^*^Two or more hospital admissions in 6 months, three or more ED visits in 6 months, or two or more ED visits in 30 day

^†^Wilcoxon rank sum (paired)

To increase recruitment at program outset, some referred patients either did not meet all of the presented utilization enrollment criteria, met them based on reported utilization at institutions outside of MSH, or were enrolled based on PACT transitions team criteria only. For the 77 patients that did not meet strict utilization criteria (Table 3), reductions in utilization were seen in hospital admissions only, where pre- to post-enrollment 6-month mean hospitalizations dropped from 0.7 to 0.5 (*p* < 0.01) and 12-month means from 1.1 to 0.9 (*p* < 0.01). Significant reductions in ED visit rates were not observed.

**Table 3.** Mean Utilization Rates of Those not Meeting Utilization Enrollment Criteria^*^ From Enrollment Date (September 2012 to October 2013) *N* = 77

Time	Location	Pre- Enrollment Mean (SD)	Post-Enrollment Mean (SD)	Percent Reduction	*p* value^†^
6 months	Hospital Admissions	0.7 (0.4)	0.5 (1.2)	29 %	<** 0.01**
	Emergency Visits	0.4 (0.7)	0.4 (0.8)	–	0.49
12 months	Hospital Admissions	1.1 (0.9)	0.9 (1.6)	18 %	< **0.01**
	Emergency Visits	0.9 (1.5)	0.9 (1.7)	–	1.0

^*^Two or more hospital admissions in 6 months

^†^Wilcoxon rank sum (paired)

### Challenges and Future Plans

The PACT Clinic serves the MSH super-utilizer population with a goal of improving quality of care and health outcomes. There are several unique aspects of the clinic that serve these goals, including multidisciplinary team-based care with the clinician/social worker unit, high level of continuity, low patient–provider ratios, open access flexibility, and intensive social support. The model is currently expanding to other sites within the Mount Sinai Health System.

Patient selection has major implications for program value; certain patients have utilization patterns that are less amenable to even intense intervention, while others may benefit from strategies that are not labor or cost intensive. The Camden Coalition described by Miller defines patients primarily by utilization profile,^13^ while others such as the Guided Care program reported by Boult, et al., rely primarily on comorbidity risk score.^3^ The PACT Clinic uses a hybrid approach of risk score, utilization history, and provider referrals, which increases patient capture. As the PACT Clinic evolves, one of the keys to its success will lie in selecting the most appropriate patients for the program.

Structurally, most care management programs in the literature have used nurse- or social worker-led teams to support existing primary care services, and have demonstrated a range of utilization results.^5^,^6^,^14^–^18^ The Aetna and Nova Health Collaboration used advanced practice nurse care coordinators, and showed 45 % lower rate of hospital admissions compared to risk-adjusted controls,^4^ while Guided Care, where specially trained nurses provided care management for Medicare patients in the top HCC risk score quartile, resulted in a nonsignificant 6 % reduction in hospital admissions and did not show a difference in ED visit rates in a cluster randomized trial.^14^ In a recent meta-analysis of strategies to reduce use of acute care services with a median of 12 months follow-up, 29 of the 36 reviewed studies used case management, and 21 of the 36 used team changes. Compared trials showed no overall difference in ED visits between intervention and control groups at 9 months, though there was a significantly reduced rate of hospital admissions (RR 0.81).^8^ Few programs that have created specialized primary care clinics such as PACT Clinic for this high-risk group have been described or evaluated in the literature to date.

This paper is intended to describe a novel program of primary care for super-utilizers, rather than rigorously test its impact on care. Conclusions regarding the magnitude of impact of the PACT Clinic should be interpreted with caution as the evaluation lacked a concurrent control group, thus raising the possibility that some of the program’s impact is attributable to regression to the mean. High-risk patients are often enrolled in services during periods of acute crisis, and utilization can decline regardless of intervention. Moreover, we could have under-detected hospitalizations and ED visits since we did not have access to data from hospitals other than MSH. Nonetheless, the preliminary data we present suggests that this is a promising model of care and is deserving of further study. We are currently conducting a comparison of PACT Clinic patients to a cohort of propensity score matched control patients.

Determining the best way to improve care for super-utilizers remains a challenge and a work in progress. In the meantime, the shift from fee-for-service payment to capitation and population management should incentivize high-value and high-quality care. There may not be one “best” way to improve care for super-utilizers, so demonstrating innovative program models in disparate populations is important to help systems make choices for their own settings. Differing levels of primary care intensity may be one way to meet the triple aim of reduced cost, improved patient experience and improved population health.^19^

## TEACHING COMMENTARY

By Sunil Kripalani, MD, MSc

### Synopsis

This article describes the implementation of the Preventable Admissions Care Team (PACT) Clinic, which uses team-based primary care to provide high-intensity medical, psychosocial, and care coordination services to “super-utilizers.” The authors provide a good description of the rationale for the program, as well as details about the setting, team structure, administrative resources, panel size, visit length, and frequency of contact. Such information is important for comparison to other programs,^20^,^21^ and for possible replication.

To evaluate the effect of the PACT Clinic, the authors performed an analysis of health care utilization in the 6–12 months before and after clinic enrollment. In this commentary, I describe two challenges in evaluating the program—co-intervention and regression to the mean.

### Co-Intervention

Co-intervention refers to any intervention other than the one being studied, and may cause bias in the outcome assessment.^22^ Co-intervention is considered most often in controlled trials, where we hope that treatment and control groups are treated similarly, apart from the intervention being studied. Indeed, a major rationale behind blinding is to help ensure this occurs.^23^ In pre–post evaluation studies such as this one, co-intervention is a consideration because care processes may evolve over time, or additional interventions may be implemented. It would be useful to know whether the health system rolled out other interventions during the study time frame, such as additional monitoring or a medication assistance program, which could affect readmission rates.

We should also be mindful of the potential for co-intervention in this study because of the way in which patients were referred to the PACT Clinic. The majority (79 %) were referred “from an inpatient transitional care program built to reduce 30-day hospital readmission rates.” It is unclear what co-intervention, if any, these patients may have received. Often, an inpatient readmission reduction program will include attention to individual patient risk factors, care coordination, medication reconciliation, patient education, and a post-discharge phone call. This bundle of services could be expected to reduce health care utilization by 25–30 %.^24^ If such services were provided, they could be partly responsible for the reduction in health care utilization attributed to the PACT Clinic.

### Regression to the Mean

Regression to the mean (RTM) is a major concern in evaluating super-utilizer interventions,^21^ which the authors acknowledge in the limitations. RTM refers to the tendency of a variable with an extreme value to be closer to the norm on subsequent measurement. We can think of measured values as reflecting the true value +/− an element of chance, variation, or measurement error that has a potential range. When an extremely high (or low) value is measured, it is likely that this chance element is at the high (or low) end of its potential range. If the measurement is repeated, it is unlikely that the chance element will be extreme a second time; it is more likely to be closer to the expected mean.^25^ The phenomenon explains why an extremely high blood pressure value is usually followed by a lower reading, absent any intervention.^26^

RTM occurs in diverse situations that involve repeated assessment. A professional athlete who has a great start to the season and lands a magazine cover probably won’t continue that level of performance in the next few games. (This is actually known as the “*Sports Illustrated* cover jinx.”^27^) RTM also helps explain why most movie sequels don’t quite live up to the original (think *Caddyshack*, *The Hangover*, *The Matrix*, etc.).^28^

In the case of super-utilizers, patients who have the highest levels of health care utilization during one year will likely have less utilization the next year.^21^ Recently, this effect was quantified in an observational study by Johnson and colleagues, who followed approximately 1650 patients identified as super-utilizers. Within 7 months, fewer than half continued to meet super-utilizer criteria, and 12 months later, only 28 % met criteria.^29^

#### Addressing RTM in Study Design

RTM is a critical factor to keep in mind when designing and evaluating quality improvement (QI) initiatives,^30^ particularly because targets for intervention are often selected on the basis of their performance being much higher or lower than that of others. RTM can be addressed in multiple ways. Statistical techniques are available to estimate the magnitude of RTM and adjust for it to some extent.^25^,^31^ A more common approach (as planned by the authors) is to retrospectively define a matched cohort of patients that can serve as a control group. In the remainder of this commentary, I will describe ways in which QI studies can be designed to reduce the potential for bias associated with RTM, and some of the real-world pressures that may challenge the implementation of these study designs.

The most rigorous approach is to use a prospective study design that includes a concurrent control group that is similar to the intervention group—the prime example being a randomized controlled trial (RCT). In the PACT initiative, if patients identified as super-utilizers were randomized to receive the intervention or usual care, then both sets of patients would have experienced similar RTM. The effect of the intervention could then be isolated by comparing the subsequent utilization of the intervention patients to that of the control group. A number of challenges would need to be addressed in order to successfully implement an RCT in this context. At an institutional level, there may be a desire for maximal program impact by intervening with all eligible super-utilizer patients rather than only half of them. At a provider level, well-intentioned referrals to the PACT Clinic take on a different tone and would likely decline knowing that only half of patients will receive clinic services. At a patient level, an RCT would require informed consent. These challenges and others could all be addressed, but they require additional up-front buy-in from the stakeholders, as well as infrastructure to manage the study.

A second design approach involves collecting additional baseline data.^28^ After defining the eligible sample on the basis of initial measurements, additional baseline data are collected for analytic purposes (i.e., the measurements that qualified patients for the study are not used as the baseline). This approach reduces evaluation bias by allowing some RTM to occur before the intervention period begins. This form of pre–post study design may be easier to implement in practice compared to an RCT, but it offers challenges as well. As applied to the PACT Clinic, this approach would involve defining the eligible sample with high utilization, observing their utilization for an additional 6–12 months, and then beginning the PACT intervention. From a practical standpoint, delaying the initial clinic appointment could lead to provider dissatisfaction as well as patient attrition. This approach would be more feasible in a different context (e.g., a hypertension study) when the additional baseline data could be collected over a shorter period of time.

A delayed intervention study design effectively combines the two above approaches. In this case, patients referred to the PACT Clinic would be randomly assigned to begin immediately, or after a period of 6–12 months. This approach allows for eventual inclusion of everyone in the targeted sample. The randomly selected delayed intervention group effectively serves as a concurrent control for the immediate intervention group. Moreover, the delayed intervention group receives an additional period of baseline assessment, so when its outcomes are assessed in a pre–post fashion, this group has had an opportunity for RTM before intervention effects are measured. This study design would be compatible with a program like PACT that is still developing and may not have the capacity to serve all eligible patients immediately; some patients could be randomized to receive immediate referral, and others scheduled to start at a later date.

An expanded form of a two-group delayed intervention design is a stepped wedge (or multiple baseline) design, which divides the target sample into multiple groups and randomly assigns them to successive roll-out stages.^32^ Again, everyone eventually receives the intervention, but those that have not received it yet serve as controls in the meantime. Multiple pre–post comparisons can also be made, as each group will have a baseline and intervention period. While this design could be employed in a patient-level intervention like PACT, it is more commonly used when randomization can occur among units of a health care institution (e.g., nursing units or clinics), which are assigned to begin an intervention at successive times. A delayed intervention design has practical advantages as well, because the QI resources needed to support an initial-roll out can be applied to the different units in succession. It is therefore well-suited to studying implementation of QI initiatives in practice.^33^

### Conclusion

Leaders of quality improvement initiatives commonly experience pressures to move quickly, expand the program’s reach, and maximize its impact. Developing a rigorous evaluation strategy in this context can be challenging. With advance planning, controlled study designs can be employed in the real-world setting, which facilitate a true understanding of a program’s effectiveness.
